# Factors associated with quality of life in patients with kidney failure managed conservatively and with dialysis: a cross-sectional study

**DOI:** 10.1186/s12882-023-03355-3

**Published:** 2023-10-27

**Authors:** Sarah So, Mark A Brown, Kelly Li

**Affiliations:** 1 Department of Renal Medicine, Nepean Hospital, Derby Street, Kingswood, 2747 NSW UK; 2 The University of Sydney, Sydney, Australia; 3 Department of Renal Medicine, St George Hospital, Kogarah, Sydney, Australia; 4 The University of New South Wales, Sydney, Australia

**Keywords:** Chronic kidney disease (CKD), Patient-reported outcomes (PROs), Health-related quality of life (HRQOL), End stage renal disease (ESRD), Kidney failure, Health status, Symptom burden, Kidney supportive care, Conservative kidney management

## Abstract

**Background:**

Later stage chronic kidney disease (CKD) is associated with poorer self-perceived health-related quality of life (HRQOL), a major consideration for many patients. Psychological factors such as depression and anxiety have been linked with poorer HRQOL. We aimed to determine if anxiety or depressive symptoms are significantly associated with self-perceived health-related quality of life, in patients with CKD Stage 5. The secondary aim was to determine which patient-associated factors are associated with HRQOL in patients with CKD Stage 5.

**Methods:**

This retrospective cross-sectional study included patients that attended the St George Hospital Kidney Supportive Care (KSC) clinic between 1 and 2015 and 30 June 2022 with CKD Stage 5 (either conservatively-managed or receiving dialysis). Patients completed surveys of their functional ‘domains’ and quality of life (EQ-5D-5L) and symptom surveys (IPOS-Renal) at their first visit. We performed multivariable linear regression analysis with the outcome of interest being HRQOL, measured using the EQ-VAS, a continuous 100-point scale, for patients undergoing conservative management or dialysis. Pre-specified variables included age, sex, eGFR (for those conservatively-managed), “feeling depressed” (IPOS-Renal), “feeling anxious” (IPOS-Renal) and “anxiety/depression” (EQ-5D-5L).

**Results:**

We included 339 patients. 216 patients received conservative kidney management (CKM) and 123 patients received dialysis. Patients receiving CKM were significantly older than those on dialysis, (median age 83 years vs. 73 years, p < 0.001). For conservatively-managed patients, variables independently associated with poorer EQ-VAS were difficulty performing usual activities (EQ-5D-5L), drowsiness (IPOS-Renal) and shortness of breath (IPOS-Renal). For patients receiving dialysis, variables that were independently associated with poorer EQ-VAS were reduced ability to perform self-care (EQ-5D-5L) and lack of energy (IPOS-Renal). Anxiety and depressive symptoms were not significantly associated with poorer EQ-VAS for either group of patients.

**Conclusions:**

Symptoms associated with reduced HRQOL include shortness of breath, drowsiness and impaired functional ability. Optimization of multidisciplinary teams focusing on these issues are likely to be of benefit.

**Supplementary Information:**

The online version contains supplementary material available at 10.1186/s12882-023-03355-3.

## Introduction

Chronic kidney disease (CKD) affects over 10% of the general population worldwide, with a higher incidence in older populations [1, 2]. As patients approach CKD Stage 5, or end-stage kidney failure, they are faced with the decision of whether to proceed with dialysis or conservative, non-dialysis, kidney management (CKM). Dialysis does not fully replace all functions of a kidney and can reduce quality of life [3], particularly in those aged over 75 years [4]. Furthermore, dialysis may not even offer a survival advantage for those aged 80 years or over, and if patients are aged 70 years or over with comorbid burden, the survival advantage is significantly reduced [5–7]. Some patients also consider quality of life, such as maintaining independence and avoiding hospitalisation to be more important than length of life [8–10]. Non-dialysis, or conservative kidney management (CKM), can offer advantages with regards to quality of life, reduced hospitalisations and enabling preferred place of death [11, 12]. As a result, the number of patients with kidney failure who elect for CKM has increased in the last decade in Australia alone [13].

Regardless of whether patients proceed down the dialysis or CKM pathway, late-stage CKD is associated with poorer self-perceived health-related quality of life (HRQOL [14, 15]). In the non-dialysis population, socioeconomic factors such as female sex and lower education [3, 16, 17], medical factors such as diabetes mellitus, vascular disease and congestive heart failure [16, 18] and anaemia [18–20] and psychological factors such as depression and anxiety [21–23] have been linked with poorer HRQOL. Although being on dialysis is independently associated with lower HRQOL [3], compared to those with CKD not on dialysis, studies that focus on predictors of poorer HRQOL in the dialysis population only include small numbers, although somesuggest a relationship between anxiety and depressive symptoms and HRQOL for patients on dialysis [24–29].

Many patients with kidney failure, whether they are managed with CKM or with dialysis, have a high symptom burden and poorer HRQOL [30, 31]. Patients’ experiences of disease and significant contributors to HRQOL should be recognised as an important area for healthcare providers to identify, understand and target.

The primary aim of this study was to determine if anxiety or depressive symptoms are significantly associated with self-perceived health-related quality of life, in patients with kidney failure (either receiving CKM or dialysis) The primary hypothesis is that self-reported anxiety and/or depressive symptoms are significantly associated with self-perceived health-related quality of life in patients with kidney failure.

The secondary aim was to determine which patient-associated factors (such as comorbidities, symptoms or biochemical parameters) are associated with HRQOL in patients with kidney failure (either receiving CKM or receiving dialysis).

## Methods

### Study design

This study is designed as a retrospective cross-sectional study.

### Setting

Patients who attend the St George Hospital Kidney Supportive Care (KSC) clinic complete surveys using a validated tools of their functional ‘domains’ and quality of life (EQ-5D-5L [32] and symptom surveys (IPOS-Renal [33] at the beginning of each clinic visit. All CKM patients are referred to the KSC clinic whilst dialysis patients are referred to the KSC clinic at the discretion of their nephrologist, common reasons being management of physical or psychological symptoms, and advance care planning. Consent is obtained for the use of this data for future research purposes at the time of collection. This study received approval from the South Eastern Sydney Local Health District Human Research Ethics Committee.

### Data collection

For each patient, demographic data, comorbidities and biochemical data were obtained from the electronic medical records system and entered into the KSC database at each clinic visit with corresponding EQ-5D-5L and IPOS-Renal surveys. Demographic data includes age at study entry, sex, date of birth, country of birth, highest level of education, primary renal disease), functional assessment (Karnofsky score [34], renal function as measured by eGFR (for patients not on dialysis), Charlson comorbidity score [35] and co-morbidities (myocardial infarction, congestive heart failure, peripheral vascular disease, cerebrovascular disease, dementia, COPD, connective tissue disease, peptic ulcer disease, diabetes mellitus, hemiplegia, leukaemia, malignant lymphoma, solid tumour, liver disease and AIDS), and biochemical data (haemoglobin, urea, creatinine, eGFR, serum albumin, corrected calcium, calcium, phosphate). Symptoms assessed on the IPOS-Renal survey include pain, shortness of breath, lack of energy, nausea, vomiting, poor appetite, constipation, mouth/oral symptoms, drowsiness, poor mobility, itching, difficulty sleeping, restless legs, skin changes, diarrhoea, taste changes, anxiety, depression, total symptom score and whether an additional concern was present that was not covered. ‘Domains’ assessed by the EQ-5D-5L survey included mobility, ability to perform self-care (personal care), ability to perform usual activities (work or hobbies), pain or discomfort, and anxiety or depression. Symptom and domain variables are answered on a severity scale from 1 (symptom not present), 2 (mild), 3 (moderate), 4 (severe) and 5 (overwhelming). However, for the purpose of this study, we have regrouped the five variables into three groups (symptom not present, mild/moderate and severe/overwhelming), for clinical relevance and ease of interpretation.

### Participants

All patients from St George and Sutherland Hospitals attending the Kidney Supportive Care (KSC) clinic between 1 and 2015 and 30 June 2022 were asked to complete the EQ-5D-5L and IPOS-Renal survey at their first KSC visit, Patients with CKD Stage 5 managed with either CKM or dialysis were included in the study. Patients were excluded if they did not have CKD Stage 5 or if they had a kidney transplant.

### Statistical methods

Primary outcome is the patients’ self-perceived health-related quality of life score, which will now be referred to as the EQ-VAS, on a continuous 100-point scale (collected on the EQ-5D-5L survey).

Data to be analysed was from participants’ first clinic visit.

Potential variables analysed included demographic data, functional assessment (Karnofsky score), eGFR (for patients not on dialysis), Charlson comorbidity score and co-morbidities and biochemical data (severity of symptoms and functional ‘domains’ from the IPOS-Renal and EQ-5D-5L survey. Prespecified variables to be analysed included anxiety and depression (on the IPOS-Renal survey) and ‘anxiety or depression’ (as a domain on the EQ-5D-5L).

Descriptive data was reported separately based on pathway (CKM or dialysis). Continuous variables were summarised with means ± SD for normally distributed variables and medians (interquartile range, IQR) for non-normally distributed variables. Unpaired t-tests were performed to compare differences in normally-distributed continuous variables between the two pathways. Mann-Whitney U tests were performed to compare differences in non-normally distributed continuous variables between the two pathways. Chi-squared tests were performed to compare differences in categorical variables between the two pathways.

Multivariable analyses were performed separately based on pathway (CKM or dialysis). For each pathway, univariable analyses were first performed for each dependent variable for the outcome of interest (EQ-VAS). Only variables with a p < 0.20 on univariable analysis were included in the multivariable model. Multivariable linear regression analysis was performed with pre-specified variables including age, sex, eGFR (for those on CKM), “Feeling depressed,” (IPOS-Renal) “Feeling anxious” (IPOS-Renal) and “Anxiety/depression” (EQ-5D-5L). Missing variable data was excluded from analysis. Statistical analyses were performed with IBM SPSS Statistics V26.0. p-values of < 0.05 were regarded as significant. The reporting of this study adheres to the STROBE checklist. This data has not been previously published.

## Results

### Patient characteristics

A total of 339 patients (216 patients on CKM and 123 patients on dialysis) were included in the study. The majority of patients on dialysis were receiving haemodialysis (85%). Patients receiving CKM were significantly older than those on dialysis (median age 83 years vs. 73 years, p < 0.001). Overall, proportions of men and women were similar between the CKM and dialysis groups. In the dialysis group, a significantly higher proportion of patients had kidney failure caused by diabetes mellitus or glomerulonephritides. In the CKM group, a significantly higher proportion of patients had kidney failure caused by ischemic nephrosclerosis. Median eGFR for patients on the CKM pathway was 14 mL/min, with a median creatinine at first clinic visit of 310 umol/L. The CKM group also had a higher proportion of comorbidities including congestive cardiac failure, dementia, a previous cerebrovascular accident and those who had smoked within the last 5 years (Table 1).

**Table 1:** Baseline characteristics at first Kidney Supportive Care Clinic visit

Variable	Conservative N = 216	On dialysis N = 123	p-value
**Age** **(median, IQR, years)**	83 (78–87)	73 (64–80)	< 0.001*
**Male sex (n, %)**	128 (59.3%)	76 (61.8%)	0.65
**Dialysis modality (n, %)**	N/A		N/A
Haemodialysis		105 (85.4%)	
Peritoneal dialysis		18 (14.6%)	
**Region of birth**			0.42
Australia/New Zealand	86 (41.0%)	59 (48.0%)	
Asia	31 (14.8%)	10 (8.1%)	
South Asia	8 (3.8%)	2 (1.6%)	
South America	2 (1.0%)	1 (0.8%)	
North America	1 (0.5%)	0 (0%)	
Middle East	17 (8.1%)	13 (10.6%)	
Western Europe	18 (8.6%)	8 (6.5%)	
Eastern Europe	39 (18.6%)	21 (17.1%)	
Pacific Islands	6 (2.9%)	5 (4.1%)	
Africa and Mauritius	2 (1.0%)	4 (3.3%)	
**Highest Level of Education**			0.37
No formal	2 (3.4%)	2 (2.2%)	
Primary	11 (18.6%)	11 (11.8%)	
Some High School	12 (20.3%)	12 (12.9%)	
Completed High School	12 (20.3%)	28 (30.1%)	
Diploma/TAFE	10 (16.9%)	24 (25.8%)	
Completed university	12 (20.3%)	16 (17.2%)	
**Primary diagnosis**			< 0.001*
Diabetes mellitus	51 (23.6%)	46 (37.4%)	
Ischemic nephrosclerosis	62 (28.7%)	15 (12.2%)	
Glomerulonephritides	13 (6.0%)	25 (20.3%)	
PCKD	4 (1.9%)	5 (4.1%)	
Malignancy (including Haematological)	6 (2.8%)	3 (2.4%)	
Interstitial nephritis	1 (0.5%)	4 (3.3%)	
Toxins	2 (0.9%)	6 (4.9%)	
Reflux	2 (0.9%)	1 (0.8%)	
Obstructive	7 (3.2%)	1 (0.8%)	
Other	5 (2.3%)	3 (2.4%)	
Unknown	63 (29.2%)	14 (11.4%)	
**Ethnicity**			0.009*
Caucasian	148 (68.5%)	92 (77.3%)	
Asian	38 (17.6%)	9 (7.6%)	
Arabic	10 (4.6%)	9 (7.6%)	
Pacific Islander	2 (0.9%)	6 (5.0%)	
ATSI	0 (0%)	0 (0%)	
Other	2 (0.9%)	3 (2.5%)	
**GFR (median, IQR, mL/min/1.73m** ^**2**^ **)**	14 (10–18)	N/A	N/A
**Urea (median, IQR, mmol/L)**	24 (18–32)	N/A	N/A
**Creatinine (median, IQR, umol/L)**	310 (242–424)	N/A	N/A
**Haemoglobin** **(mean ± SD, g/L)**	107 ± 15	109 ± 14	0.95
**Potassium (mean ± SD, mmol/L)**	4.7 ± 0.6	4.8 ± 0.47	0.90
**Corrected calcium (mean ± SD, mmol/L)**	2.33 ± 0.19	2.41 ± 0.17	0.53
**Phosphate (mean ± SD, mmol/L)**	1.56 ± 0.46	1.53 ± 0.47	0.21
**Albumin (mean ± SD, g/L)**	34 ± 6	31 ± 6	0.53
**PTH (median, IQR, pmol/L)**	16 (9–26)	16 (11–34)	0.52
**Karnofsky scale (mean ± SD)**	65 ± 15	71 ± 14	0.28
**BMI (mean + SD, kg/m** ^**2**^ **)**	27 ± 6	27 ± 8	0.42
**Time on dialysis (median, IQR, months)**	N/A	19 (3–52)	N/A
**Charlson comorbidity score (median, IQR)**	5 (3–7)	5 (3–6)	0.051
**Comorbidities**			
Myocardial infarct	9 (4.2%)	3 (2.4%)	0.41
Congestive cardiac failure	65 (30.1%)	17 (13.8%)	0.001*
Peripheral vascular disease	25 (11.6%)	15 (12.2%)	0.87
Previous CVA	26 (12.0%)	6 (4.9%)	0.03*
Dementia	14 (6.5%)	0 (0%)	0.004*
COPD	37 (17.1%)	18 (14.6%)	0.55
Peptic ulcer disease	5 (2.3%)	2 (1.6%)	0.67
**Diabetes mellitus**	122 (56.5%)	69 (56.1%)	0.95
Leukaemia	2 (0.9%)	2 (1.6%)	0.57
Malignant lymphoma	8 (3.7%)	2 (1.6%)	0.28
Solid tumour (non-metastatic)	33 (15.3%)	14 (11.4%)	0.32
Metastatic solid tumour	15 (6.9%)	6 (4.9%)	0.45
Mild liver disease	5 (2.3%)	2 (1.6%)	0.67
Moderate to severe liver disease	4 (1.9%)	0 (0%)	0.13
**Smoked within the last 5 years**	187 (86.6%)	89 (55.2%)	0.001*

*p values that are significant

Urea and creatinine were not added into the table for haemodialysis and peritoneal dialysis patients as they were on dialysis at the time of their first KSC visit

### Symptom data

Baseline IPOS-Renal and EQ-5D-5L data are presented in Tables 2 and 3. A significantly higher proportion of patients on dialysis reported pain and restless legs compared to patients receiving CKM, on the IPOS-Renal survey. For EQ-5D-5L functional domains, there was no significant difference between mobility, ability to provide self-care, ability to undertake usual activities, pain/discomfort or anxiety/depression between the two pathways. There was no significant difference in EQ-VAS between the two pathways.

**Table 2:** Baseline symptom data (IPOS-Renal)

Variable	Conservative N = 216	On dialysis N = 123	p-value
**Pain (n, %)**			0.013*
Not at all	86 (42.0%)	42 (36.2%)	
Slight/moderate	80 (39.0%)	35 (30.2%)	
Severe/overwhelming	39 (19.0%)	39 (33.6%)	
**Shortness of breath (n,%)**			0.35
Not at all	88 (43.3%)	60 (51.7%)	
Slight/moderate	90 (44.3%)	44 (37.9%)	
Severe/overwhelming	25 (12.3%)	12 (10.3%)	
**Lack of energy (n, %)**			
Not at all	39 (19.0%)	15 (12.9%)	0.27
Slight/moderate	102 (49.8%)	67 (57.8%)	
Severe/overwhelming	64 (31.2%)	34 (29.3%)	
**Nausea (n, %)**			0.30
Not at all	160 (77.3%)	84 (72.4%)	
Slight/moderate	38 (18.4%)	29 (25.0%)	
Severe/overwhelming	9 (4.3%)	3 (2.6%)	
**Vomiting (n, %)**			0.49
Not at all	188 (91.7%)	103 (88.8%)	
Slight/moderate	14 (6.8%)	12 (10.3%)	
Severe/overwhelming	3 (1.5%)	1 (0.9%)	
**Poor appetite (n, %)**			0.85
Not at all	107 (52.2%)	64 (55.2%)	
Slight/moderate	66 (32.2%)	36 (31.0%)	
Severe/overwhelming	32 (15.6%)	16 (13.8%)	
**Constipation (n, %)**			0.88
Not at all	129 (62.9%)	71 (61.2%)	
Slight/moderate	62 (30.2%)	38 (32.8%)	
Severe/overwhelming	14 (6.8%)	7 (6.0%)	
**Mouth problems (n, %)**			0.10
Not at all	82 (41.0%)	56 (48.7%)	
Slight/moderate	84 (42.0%)	49 (42.6%)	
Severe/overwhelming	34 (17.0%)	10 (8.7%)	
**Drowsiness (n, %)**			0.52
Not at all	66 (32.5%)	41 (35.3%)	
Slight/moderate	99 (48.8%)	59 (50.9%)	
Severe/overwhelming	38 (18.7%)	16 (13.8%)	
**Poor mobility (n, %)**			0.48
Not at all	52 (25.4%)	36 (31.6%)	
Slight/moderate	94 (45.9%)	49 (43.0%)	
Severe/overwhelming	59 (28.8%)	29 (25.4%)	
**Itching (n, %)**			
Not at all	84 (40.6%)	42 (36.5%)	0.55
Slight/moderate	83 (40.1%)	45 (39.1%)	
Severe/overwhelming	40 (19.3%)	28 (24.3%)	
**Difficulty sleeping (n, %)**			0.05
Not at all	89 (43.2%)	38 (33.0%)	
Slight/moderate	79 (38.3%)	43 (37.4%)	
Severe/overwhelming	38 (18.4%)	34 (29.6%)	
**Restless legs (n, %)**			< 0.001*
Not at all	152 (75.2%)	62 (53.4%)	
Slight/moderate	40 (19.8%)	39 (33.6%)	
Severe/overwhelming	10 (5.0%)	15 (12.9%)	
**Skin changes (n, %)**			0.70
Not at all	133 (64.3%)	70 (60.3%)	
Slight/moderate	62 (30.0%)	37 (31.9%)	
Severe/overwhelming	12 (5.8%)	9 (7.8%)	
**Diarrhoea (n, %)**			0.86
Not at all	165 (80.5%)	90 (78.3%)	
Slight/moderate	30 (14.6%)	18 (15.7%)	
Severe/overwhelming	10 (4.9%)	7 (6.1%)	
**Taste changes**			0.37
Not at all	93 (64.1%)	48 (60.8%)	
Slight/moderate	37 (25.5%)	26 (32.9%)	
Severe/overwhelming	15 (10.3%)	5 (6.3%)	
**Additional concern present (n, %)**	39 (18.1%)	22 (17.9%)	0.97
**Feeling anxious (n, %)**			0.12
Not at all	80 (41.7%)	35 (33.7%)	
Slight/moderate	76 (39.6%)	39 (37.5%)	
Severe/overwhelming	36 (18.8%)	30 (28.8%)	
**Feeling depressed (n, %)**			0.52
Not at all	96 (50.8%)	46 (45.1%)	
Slight/moderate	71 (37.6%)	40 (39.2%)	
Severe/overwhelming	22 (11.6%)	16 (15.7%)	

*p values that are significant

**Table 3:** Baseline functional and symptom domain data (EQ-5D-5L)

Variable	Conservative	On dialysis	p-value
**Mobility (n, %)**			
No problems	27 (21.8%)	14 (25.5%)	0.85
Slight/moderate problems	63 (50.8%)	26 (47.3%)	
Severe problems/unable to perform	34 (27.4%)	15 (27.3%)	
**Self-care (n, %)**			0.19
No problems	72 (57.6%)	36 (65.5%)	
Slight/moderate problems	31 (24.8%)	15 (27.3%)	
Severe problems/unable to perform	22 (17.6%)	4 (7.3%)	
**Usual activities (n, %)**			0.17
No problems	39 (31.7%)	14 (25.5%)	
Slight/moderate problems	40 (32.5%)	26 (47.3%)	
Severe problems/unable to perform	44 (35.8%)	15 (27.3%)	
**Pain/discomfort (n, %)**			0.46
No pain	51 (41.1%)	22 (40.0%)	
Slight/moderate problems	51 (41.1%)	19 (34.5%)	
Severe problems/overwhelming	22 (17.7%)	14 (25.5%)	
**Anxiety/depression (n, %)**			0.79
Not anxious/depressed	54 (43.5%)	22 (40.0%)	
Slight/moderate problems	62 (50.0%)	28 (50.9%)	
Severe problems/overwhelming	8 (6.5%)	5 (9.1%)	
**Quality of life score (self-rated, median, IQR)**	52.5 (45–75)	60 (50–75)	0.24

*p values that are significant

### Multivariable analyses

#### Conservative kidney management

For the patients receiving CKM, variables significantly associated with poorer EQ-VAS on univariable analysis were region of birth (p = 0.04), Karnofsky score (p = 0.008), pain (IPOS-Renal) (p = 0.005), shortness of breath (IPOS-Renal) (p = 0.006), lack of energy (IPOS-Renal) (p = 0.003), mouth problems (IPOS-Renal) (p = 0.013), drowsiness (IPOS-Renal) (p < 0.001), poor mobility (IPOS-Renal) (p < 0.001), feeling anxious (IPOS-Renal) (p = 0.015), feeling depressed (IPOS-Renal) (p = 0.004), reduced mobility (EQ-5D-5L) (p < 0.001), reduced ability to perform self-care (EQ-5D-5L) (p < 0.001), reduced ability to perform usual activities (EQ-5D-5L) (p < 0.001), pain or discomfort (EQ-5D-5L) (p = 0.002) and anxiety or depression (EQ-5D-5L) (p = 0.005). Full results of the univariable analyses are attached in the Supplemental Material 1.

**Fig. 1:**
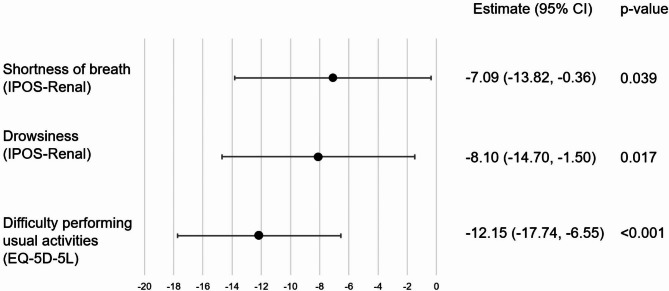
Factors associated with HRQOL in CKD Stage 5 not on dialysis

On multivariable analysis, variables that were independently associated with poorer EQ-VAS were difficulty performing usual activities (EQ-5D-5L), drowsiness (IPOS-Renal) and shortness of breath (IPOS-Renal) (Fig. 1).

#### Dialysis

For the patients receiving dialysis, variables significantly associated with poorer EQ-VAS on univariable analysis were lower Karnofsky score (p = 0.007), lack of energy (IPOS-Renal) (p = 0.01), nausea (IPOS-Renal) (p = 0.008), poor appetite (IPOS-Renal) (p = 0.04), poor mobility (IPOS-Renal) (p = 0.015), poor mobility (EQ-5D-5L) (p < 0.001), ability to perform self-care (EQ-5D-5L) (p < 0.001), ability to perform usual activities (EQ-5D-5L) (p < 0.001), pain or discomfort (EQ-5D-5L) (p = 0.043) and anxiety or depression (EQ-5D-5L) (p = 0.007). Full results of the univariable analyses are attached in the Supplemental Material 1.

**Fig. 2:**
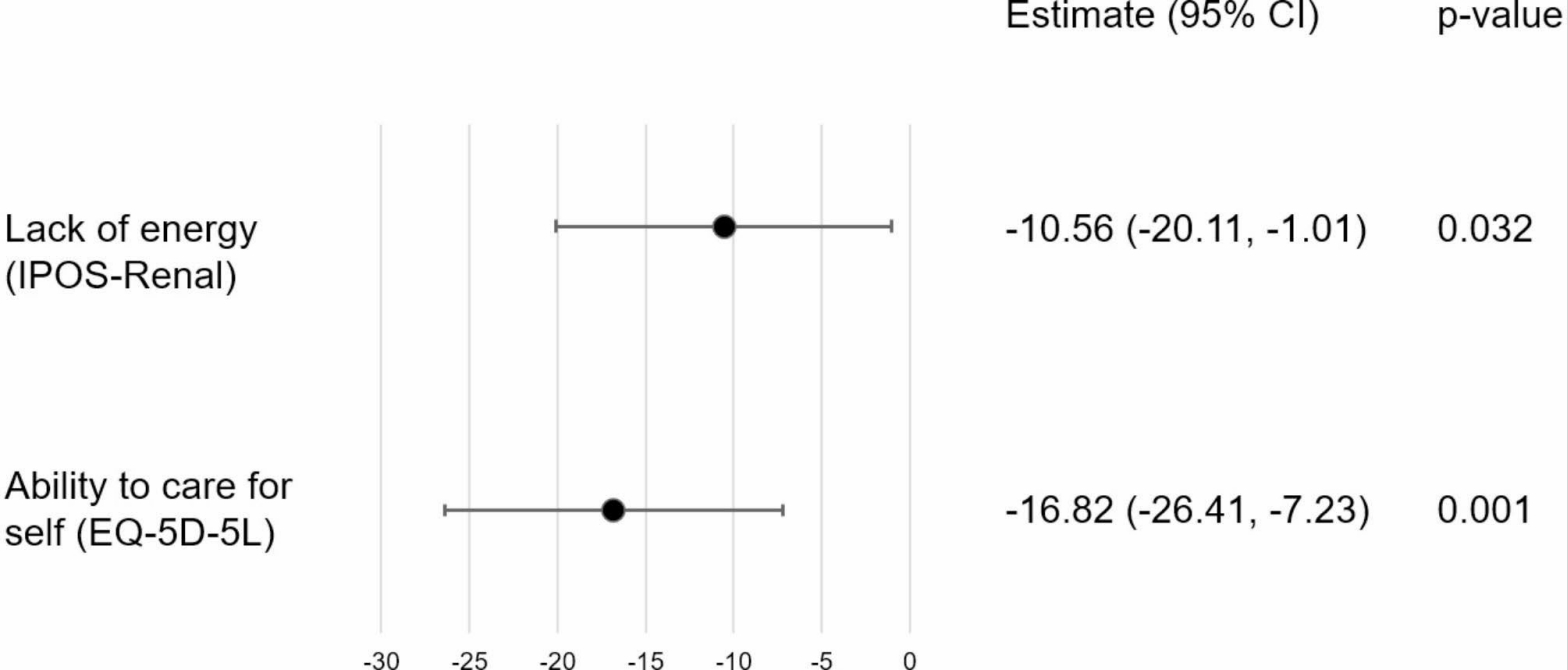
Factors associated with HRQOL in CKD Stage 5 on dialysis

On multivariable analysis, variables that were independently associated with poorer EQ-VAS were reduced ability to perform self-care (EQ-5D-5L) and lack of energy (IPOS-Renal) (Fig. 2).

## Discussion

Our findings suggest that for patients with CKD Stage 5 not receiving dialysis, independent predictors of poorer self-perceived health-related QOL (measured through the EQ-VAS) include reduced ability to perform their usual activities (work or leisure activities), higher severity of self-rated shortness of breath and higher severity of self-rated drowsiness. Interestingly, despite being significant on univariable analysis, anxiety and depressive symptoms (as measured by either the EQ-5D-5L or the IPOS-Renal surveys) were not independently associated with poorer EQ-VAS. For patients with CKD Stage 5 receiving dialysis, independent predictors of poorer EQ-VAS include inability to perform self-care (personal care, such as showering and washing) and lack of energy. Again, despite being significant on univariable analysis, anxiety and depressive symptoms were not independently associated with poorer EQ-VAS.

These findings contrast with the majority of the literature in the non-dialysis, CKD population which broadly suggests that anxiety and depressive symptoms are associated with poorer HRQOL. However, many of the studies reporting this association [21–23] in non-dialysis CKD patients are limited by including only very small numbers of patients with CKD Stage 5. In most studies, patients with CKD Stage 5 consist of less than 50 participants and findings may have thus been confounded by small numbers. Our findings are similar to one larger study [18], which included 225 patients with CKD Stage 5 not receiving dialysis, which also did not find an association between anxiety and depressive symptoms and HRQOL. Compared to previous studies, we were able to collect more detailed symptom data (IPOS-Renal) and more data on patients’ general function (EQ-5D-5L). The larger number of variables we had available may have reduced the risk of confounding compared to previous studies. However, one weakness of our study that may have contributed to our negative findings could be the use of a generic health tool, the EQ-5D-5L, rather than tools that were targeted specifically to assess anxiety or depression, although the EQ-5D-5L still displayed positive correlation with the PHQ-9 for depression and the GAD-7 for anxiety [36]. A previous study utilising IPOS-Renal [37] has also linked symptom burden to HRQOL. Surprisingly, for those receiving CKM, shortness of breath and drowsiness were the two major symptoms independently associated with poorer EQ-VAS, compared to pain. This is also the first study to our knowledge that has linked these specific symptoms to HRQOL and suggests that further research should be performed to validate these findings, as well as ensuring methods of addressing these symptoms. A major independent predictor of EQ-VAS included patients’ difficulty performing their usual activities. Therefore, an approach to improve patients’ HRQOL may be to place further emphasis on multidisciplinary involvement, including allied health colleagues to optimise patient function in their home, as recommended for CKM [38].

In the dialysis population, our findings that anxiety and depressive symptoms were not significantly associated with EQ-VAS is also in contrast to the current literature [24–29]. However, our patients reflect a subgroup of dialysis patients with relatively higher symptom burden, necessitating KSC referral and do not reflect the general dialysis population. One reason for this difference may be that most of these other studies used only one symptom survey, the SF36 survey, to collect symptom data, which does not cover the extensive range of renal-specific symptoms collected by using the IPOS-Renal survey. The published studies are also limited by including few data on patient comorbidities and these unmeasured variables (symptoms and comorbidities) could have potentially confounded their results. It was surprising that we did not find that symptoms commonly thought to contribute to HRQOL such as pain, to be significantly associated with poorer EQ-VAS. Instead, being able to perform self-care and lack of energy were the main factors linked to poorer EQ-VAS. This lends further support to optimizing multidisciplinary allied health involvement in the care of our dialysis patients, as it may impact significantly on HRQOL.

The most significant impact on self-perceived QOL in both groups was patients’ functional capacity, rather than physical symptoms (pain/discomfort) or psychological symptoms (anxiety/depression). Functional aspects are not routinely assessed in nephrology services, and it may be important to direct resources towards interventions aimed at maintaining functional capacity for patients with kidney failure.

Our study was limited by its retrospective cross-sectional nature, as we were only able to use data that was already collected. There may also be other variables that we did not collect, which may have confounded our conclusions, though the validated tools we used are fairly extensive. Although a strength of our study was its relatively large numbers compared to most of the published studies, our dialysis population was limited by only having a very small number of patients on peritoneal dialysis and by being a selected group who were referred to the KSC clinic for additional care. Further research will need to be conducted for patients of other cultural and logistical settings.

## Conclusion

For patients with kidney failure managed without dialysis, factors that are significantly associated with poor HRQOL (measured by EQ-VAS) include shortness of breath and drowsiness, which have not been described in the literature before. Reduced functional ability is associated with reduced HRQOL in patients with CKD Stage 5 managed with or without dialysis, and optimization of multidisciplinary teams within KSC units are likely to be of benefit. Further research is needed to validate these findings and to determine whether allied heath interventions can improve HRQOL in these patients.

### Electronic supplementary material

Below is the link to the electronic supplementary material.

Supplementary Material 1

## Data Availability

The datasets used and analysed during the current study are available from the corresponding author on reasonable request.
