# Supplementary material for: Factors associated with quality of life in patients with kidney failure managed conservatively and with dialysis: a cross-sectional study
Source: BMC Nephrol. 2023 Oct 27;24:322. doi: 10.1186/s12882-023-03355-3 (PMC10605777; doi:10.1186/s12882-023-03355-3)
Supplement: Supplementary file 1 — Supplementary Material 1 [file 12882_2023_3355_MOESM1_ESM.docx]

**FACTORS ASSOCIATED WITH QUALITY OF LIFE IN PATIENTS WITH KIDNEY FAILURE MANAGED CONSERVATIVELY AND WITH DIALYSIS: A CROSS-SECTIONAL STUDY**

**SUPPLEMENTAL MATERIAL**

**CONSERVATIVE KIDNEY MANAGEMENT**

| Independent variable | Estimate (95% CI) | p-value |
| --- | --- | --- |
| Sex | -4.12 (-13.13, 4.90) | 0.37 |
| Age at visit | 0.32 (-0.29, 0.93) | 0.31 |
| Year of first KSC visit | 0.05 (- 2.41, 2.50) | 0.97 |
| Region of birth | -1.46 (-2.85, -0.075) | 0.04* |
| Born in Australia/New Zealand | 3.67 (-5.53, 12.86) | 0.43 |
| Highest Level of Education | -0.08 (-4.98, 4.82) | 0.97 |
| Primary diagnosis | 0.16 (-0.89, 1.20) | 0.77 |
| Ethnicity | 2.62 (-3.15, 8.39) | 0.37 |
| Karnofsky score | 0.49 (0.13, 0.85) | 0.008* |
| BMI | -0.30 (-1.50, 0.90) | 0.61 |
| ESA | -10.31 (-35.66, 15.04) | 0.40 |
| Haemoglobin | 0.003 (-0.34, 0.35) | 0.99 |
| Potassium | 0.92 (-5.94, 7.78) | 0.79 |
| Urea | -0.40 (-0.87, 0.07) | 0.09 |
| Creatinine | -0.004 (-0.03, 0.02) | 0.75 |
| eGFR | 0.017 (-0.56, 0.60) | 0.95 |
| Corrected calcium | -34.79 (-72.61, 3.04) | 0.07 |
| Phosphate | 10.19 (-4.01, 24.39) | 0.16 |
| PTH | -0.24 (-0.83, 0.35) | 0.40 |
| Albumin | 0.17 (-0.72, 1.06) | 0.71 |
| Pain (IPOS Renal) | -8.57 (-14.44, -2.70) | 0.005* |
| Shortness of breath | -8.96 (-15.32, -2.59) | 0.006* |
| Lack of energy | -9.39 (-15.58, -3.20) | 0.003* |
| Nausea | -5.93 (-15.04, 3.18) | 0.20 |
| Vomiting | -0.65 (-15.67, 14.37) | 0.93 |
| Poor appetite | -3.66 (-9.66, 2.34) | 0.23 |
| Constipation | -5.60 (-13.54, 2.35) | 0.17 |
| Mouth problems | -8.11 (-14.45, -1.77) | 0.013* |
| Drowsiness | -12.01 (-18.18, -5.83) | <0.001* |
| Poor mobility | -13.12 (-18.36, -7.87) | <0.001* |
| Itching | -1.80 (-7.86, 4.26) | 0.56 |
| Difficulty sleeping | -1.99 (-7.66, 3.68) | 0.49 |
| Restless legs | -5.41 (-13.12, 2.31) | 0.17 |
| Changes in skin | -5.75 (-13.34, 1.84) | 0.14 |
| Diarrhoea | -4.89 (-12.99, 3.21) | 0.23 |
| Taste changes | -6.68 (-14.72, 1.36) | 0.10 |
| Additional concern present | 6.05 (-6.37, 18.47) | 0.34 |
| Feeling anxious | -7.38 (-13.30, -1.45) | 0.015* |
| Feeling depressed | -10.20 (-17.05, -3.35) | 0.004* |
| Mobility | -13.50 (-19.20, -7.80) | <0.001* |
| Ability to perform self-care | -10.40 (-15.95, -4.85) | <0.001* |
| Ability to perform usual activities | -13.75 (-18.51, -8.99) | <0.001* |
| Pain/discomfort | -9.18 (-14.93, -3.43) | 0.002* |
| Anxiety/depression | -10.35 (-17.53, -3.18) | 0.005* |
| Previous myocardial infarction | 17.94 (-28.18, 64.07) | 0.44 |
| Congestive cardiac failure | -9.26 (-19.10, 0.58) | 0.07 |
| Peripheral vascular disease | -4.71 (-18.30, 8.87) | 0.49 |
| Previous cerebrovascular accident | 11.94 (-1.97, 25.85) | 0.09 |
| Dementia | -0.85 (-18.84, 17.15) | 0.93 |
| COPD | -4.73 (-16.86, 7.40) | 0.44 |
| Diabetes mellitus | -3.01 (-11.91, 5.88) | 0.50 |
| Diabetes mellitus end organ damage | -4.62 (-13.44, 4.19) | 0.30 |
| Malignant lymphoma | 4.96 (-18.48, 28.4) | 0.68 |
| Solid tumour (non-metastatic) | 4.58 (-6.80, 15.95) | 0.43 |
| Metastatic solid tumour | -3.24 (-22.57, 16.10) | 0.74 |
| Mild liver disease | 5.01 (-16.05, 26.07) | 0.64 |
| Moderate to severe liver disease | -4.81 (-37.66, 28.04) | 0.77 |
| Charlson comorbidity score | -0.81 (-2.72, 1.11) | 0.41 |
| Smoked within the last 5 years | -5.94 (-18.70, 6.82) | 0.36 |

*p values that are significant

Univariable linear regression analyses were performed, with self-rated quality of life score as the dependent variable. Independent variables with insufficient data were not analysed.

**ON DIALYSIS**

| Independent variable | Estimate (95% CI) | p-value |
| --- | --- | --- |
| Sex | 7.61 (-3.53, 18.75) | 0.18 |
| Age at visit | 0.21 (-0.30, 7.11) | 0.42 |
| Dialysis modality | -6.38 (-19.07, 6.32) | 0.32 |
| Year of first KSC visit | 0.49 (-2.44, 3.41) | 0.74 |
| Region of birth | -1.25 (-2.86, 0.36) | 0.13 |
| Born in Australia/New Zealand | 8.01 (-2.25, 18.26) | 0.12 |
| Highest Level of Education | -1.15 (-5.50, 3.21) | 0.60 |
| Primary diagnosis | 0.02 (-1.41, 1.44) | 0.98 |
| Ethnicity | -0.14 (-4.35, 4.07) | 0.95 |
| Karnofsky score | 0.76 (-0.22, 1.29) | 0.007* |
| Time on dialysis (months) | 0.03 (-0.07, 0.12) | 0.58 |
| BMI | -0.95 (-28.80, 26.90) | 0.74 |
| ESA | 15 (-48.32, 78.32) | 0.51 |
| Haemoglobin | -0.34 (-0.78, 0.11) | 0.14 |
| Potassium | 0.79 (-8.69, 10.27) | 0.87 |
| Corrected calcium | -25.57 (-62.96, 11.82) | 0.17 |
| Phosphate | -9.71 (-22.29, 4.87) | 0.20 |
| PTH | 0.03 (-0.39, 0.45) | 0.88 |
| Albumin | 0.72 (-0.51, 1.95) | 0.24 |
| Pain (IPOS Renal) | -1.81 (-8.42, 4.79) | 0.58 |
| Shortness of breath | -6.86 (-14.56, 0.84) | 0.08 |
| Lack of energy | -10.37 (-18.18, -2.56) | 0.01* |
| Nausea | -12.85 (-22.25, -3.46) | 0.008* |
| Vomiting | -10.20 (-28.03, 7.64) | 0.26 |
| Poor appetite | -8.25 (-16.10, -0.39) | 0.04* |
| Constipation | -3.20 (-12.29, 5.89) | 0.48 |
| Mouth problems | -1.19 (-10.04, 7.67) | 0.79 |
| Drowsiness | -1.30 (-10.34, 7.75) | 0.77 |
| Poor mobility | -9.14 (-16.39, -1.89) | 0.015* |
| Itching | -1.82 (-9.24, 5.60) | 0.62 |
| Difficulty sleeping | -4.90 (-11.84, 2.04) | 0.16 |
| Restless legs | 0.03 (-7.88, 7.95) | 0.99 |
| Changes in skin | -4.84 (-13.91, 4.23) | 0.29 |
| Diarrhoea | -6.27 (-16.00, 3.47) | 0.20 |
| Taste changes | -8.23 (-17.58, 1.13) | 0.08 |
| Additional concern present | -8.28 (-25.89, 9.33) | 0.35 |
| Feeling anxious | -2.15 (-9.54, 5.24) | 0.56 |
| Feeling depressed | -6.63 (-14.88, 1.62) | 0.11 |
| Mobility | -14.26 (-20.16, -8.34) | <0.001* |
| Ability to perform self-care | -18.10 (-25.43, -10.78) | <0.001* |
| Ability to perform usual activities | -14.30 (-20.54, -8.06) | <0.001* |
| Pain/discomfort | -6.74 (-13.24, -0.24) | 0.043* |
| Anxiety/depression | -10.81 (-18.51, -3.11) | 0.007* |
| Congestive cardiac failure | -4.48 (-18.26, 9.31) | 0.52 |
| Peripheral vascular disease | -11.94 (-38.96, 15.08) | 0.38 |
| Previous cerebrovascular accident | -14.66 (-36.73, 7.41) | 0.19 |
| COPD | -2.52 (-20.27, 15.23) | 0.78 |
| Diabetes mellitus | -5.04 (-15.96, 5.87) | 0.36 |
| Diabetes mellitus end organ damage | -2.70 (-8.02, 2.63) | 0.31 |
| Leukaemia | -5.85 (-24.95, 13.14) | 0.54 |
| Malignant lymphoma | -0.76 (-19.82, 18.31) | 0.94 |
| Solid tumour (non-metastatic) | 0.97 (-6.28, 8.22) | 0.79 |
| Metastatic solid tumour | -0.26 (-4.80, 4.28) | 0.91 |
| Mild liver disease | -6.61 (-44.69, 31.48) | 0.73 |
| Charlson comorbidity score | -1.83 (-4.62, 0.95) | 0.19 |
| Smoked within the last 5 years | -5.40 (-17.11, 6.30) | 0.36 |

*p values that are significant

Univariable linear regression analyses were performed, with self-rated quality of life score as the dependent variable. Independent variables with insufficient data were not analysed.
